# Superwetting Stainless Steel Mesh Used for Both Immiscible Oil/Water and Surfactant-Stabilized Emulsion Separation

**DOI:** 10.3390/membranes13100808

**Published:** 2023-09-24

**Authors:** Yu-Ping Zhang, Ya-Ning Wang, Li Wan, Xin-Xin Chen, Chang-Hua Zhao

**Affiliations:** 1 College of Chemistry and Materials Engineering, Hunan University of Arts and Science, Changde 415000, China; wanli@huas.edu.cn; 2 College of Ecology and Environment, Zhengzhou University, Zhengzhou 450001, China; 202022392015755@gs.zzu.edu.cn (Y.-N.W.);; 3 College of Chemistry and Chemical Engineering, Henan Institute of Science and Technology, Xinxiang 453003, China; chenxinxin920@163.com

**Keywords:** solvothermal method, superwettability, stainless steel mesh, oil/water separation, emulsion separation

## Abstract

The design and fabrication of advanced membrane materials for versatile oil/water separation are major challenges. In this work, a superwetting stainless steel mesh (SSM) modified with in situ-grown TiO_2_ was successfully prepared via one-pot hydrothermal synthesis at 180 °C for 24 h. The modified SSM was characterized by means of scanning electron microscopy, energy spectroscopy, and X-ray photoelectron spectroscopy analysis. The resultant SSM membrane was superhydrophilic/superoleophilic in air, superoleophobic underwater, with an oil contact angle (OCA) underwater of over 150°, and superhydrophobic under oil, with a water contact angle (WCA) as high as 158°. Facile separation of immiscible light oil/water and heavy oil/water was carried out using the prewetting method with water and oil, respectively. For both “oil-blocking” and “water-blocking” membranes, the separation efficiency was greater than 98%. Also, these SSMs wrapped in TiO_2_ nanoparticles broke emulsions well, separating oil-in-water and oil-in-water emulsions with an efficiency greater than 99.0%. The as-prepared superwetting materials provided a satisfactory solution for the complicated or versatile oil/water separation.

## 1. Introduction

Due to the increasing amount of industrial oily wastewater and the numerous incidents involving oil spills, oil/water separation has become an urgent issue in modern chemical industrial processes and environmental protection [1,2]. There are three types of conventional wastewater treatment options for oily wastewater: biochemical, chemical, and physical. These processes mainly involve gravity, suspension, filtration, sedimentation, centrifugation, in situ combustion, anaerobic treatment technologies, etc. [3,4,5,6,7]. Although the aforementioned approaches are capable of water treatment, their efficiency and effectiveness are poor, they necessitate a great deal of labor, financial, and material resources, and even introduce potential contamination [8]. Membrane separation technology is desirable due to its high separation efficiency, wide range of applications, low energy consumption, a relatively simple operation, and less secondary pollution. It has been extensively utilized for the treatment of oily wastewater and is capable of simultaneously recovering pure water and oil [9]. Inspired by biological surfaces, materials with special wetting properties are attracting attention for their potential in the field of oil–water separation. In general, superhydrophobic/superoleophilic membranes are desirable for the penetration of heavy oil or the absorption of floating oil on the water surface, but underwater superoleophobic membranes with specific superhydrophobicity underoil are suitable for the separation of light oil/water and heavy oil/water, respectively.

Emulsions, as unique oil–water mixtures with small droplets, low density and high stability, have encountered many difficulties in the treatment process [10,11,12]. Conventional filtration membranes are prone to oil adhesion and contamination when separating emulsions, leading to pore blockage and failure of separation capacity [13]. By adjusting the chemical composition and roughness of the membrane surface, new membrane materials with special wettability were developed to improve separation efficiency for complex oil–water systems. Zeng et al. [14] provided a TA-ZIF-8@MXene MOF composite membrane which integrated the advantages of tannic acid nanoparticles and MXene modified ZIF-8. The prepared composite membrane has good water flux, chemical stability and ideal oil–water emulsion separation performance. Wei et al. reported a superhydrophobic/superoleophilic ceramic membrane with a micro-nano hierarchical structure. It was constructed using ZnO nanoflowers and a low-surface-energy material, n-octyltriethoxysilane, and was successfully used for water-in-oil emulsion separation [15]. Despite the obvious advantages of functional membranes with special wettability in separating emulsions, the design and fabrication of membrane materials with selective separation properties still face challenges such as high costs, complicated processes and versatile applications [16]. Oil–water separation is generally involved in the separation of immiscible light oil–water mixtures, heavy oil–water mixtures, oil-in-water emulsions and water-in-oil emulsions [17]. Membrane materials with special wettability like superhydrophobicity or superhydrophilicity/superoleophobicity underwater are usually used for simple and single oil–water separation. Establishing how to prepare a desirable membrane with versatile and flexible separation selectivity is a major challenge.

In this work, titanium dioxide was grown in situ on the etched SSM via the solvothermal method. Four types of mixtures were selected for the separation using the superamphiphilic SSMs, which were driven solely by gravity, respectively. The as-prepared superwetting SSM possessed both superhydrophilic/underwater superoleophobic and superhydrophobic properties under oil. The fabricated superwetting SSMs were not only used for the separation of the light oil/water and heavy oil/water, but also for the continuous and efficient emulsion (o/w and w/o) separation. The simple preparation strategy provided a practical way to develop inexpensive and efficient membrane materials for complicated and versatile oil/water separation.

## 2. Experimental

### 2.1. Materials

SSMs (304, 800 mesh) were bought from Anping County Kai Zhong Wire Mesh Products Co., Ltd. (Anping County, Hengshui, China). Tetrabutyl titanate (≥99%), anhydrous ethanol (GC, ≥99.8%), isooctane (AR), and hydrofluoric acid (AR) were purchased from Shanghai Aladdin Biochemical Technology Co. (Shanghai, China) Span 80 (AR, 96%), Twain 80 (AR, 99.5%), and glycerol (AR) were purchased from Hunan Huihong Reagent Co. (Changsha, China).

### 2.2. Preparation of Underwater Super Oleophobic SSM

The SSMs were immersed in HCl (0.1 mol/L) and ethanol and water in turn at room temperature and ultrasonically cleaned for 30 min to remove oxidized material, oil and other residual dirt. The pre-cleaned substrate was immersed in 20% HF and chemically etched at room temperature for about 1 h, after which it was washed with deionized water and dried via nitrogen blowing. We then prepared a 40 mL mixture of glycerol and ethanol (1:3, *v*/*v*) and stirred well. We then added 2.5 mL of tetrabutyl titanate to the above mixture, stirred well and poured the final mixture into a 50 mL Teflon-lined autoclave. After the etched SSMs were immersed in the autoclave, it was then sealed and heated to 180 °C for 24 h. After the autoclave was cooled to room temperature naturally, the SSMs were taken out and rinsed with ethanol and deionized water several times.

### 2.3. Preparation of Emulsions

Isooctane was selected as the oil phase for both surfactant-stabilized water/oil (w/o) and oil/water (o/w) emulsions. Preparation of water/oil emulsion: the volume ratio of oil to water was 50:1, we added 0.5 g of Span 80, and stirred for 10 min using a high-speed homogenizer with the rotational speed of 4500 r min^−1^. Preparation of oil/water emulsion: the volume ratio of water to oil was 50:1, we added 0.5 g of Tween 80, and stirred for 10 min using the high-speed homogenizer at 4500 r min^−1^. Both milky surfactant-stabilized emulsions prepared were highly stable for 24 h.

### 2.4. Characterization

The surface morphology of SSMs with different wettability was characterized using a field emission scanning electron microscope (FESEM, Zeiss Sigma500, Oberkochen, Germany). The content of chemical elements of the modified SSM was analyzed via EDS (BRUKER XFlash 6130, Oxford, UK). The chemical component of different processed samples was determined via X-ray photoelectron spectrometer (XPS, Thermo Scientific, Nexsa, Waltham, MA, USA). Fourier transform infrared (FT-IR, NICOLET IS5, Madison, WI, USA) was used for the characterization of the scraped coating on the as-prepared SSW surface. The feed and permeate emulsions were characterized using a biological microscope (NE600, Ningbo Yongxin Optical Co., Ningbo, China) and a laser particle size-zeta potential analyzer (Malvern Instruments Ltd., Malvern, UK).

## 3. Results and Discussion

### 3.1. Characterization of Surface Structure and Composition

**Figure 1:**
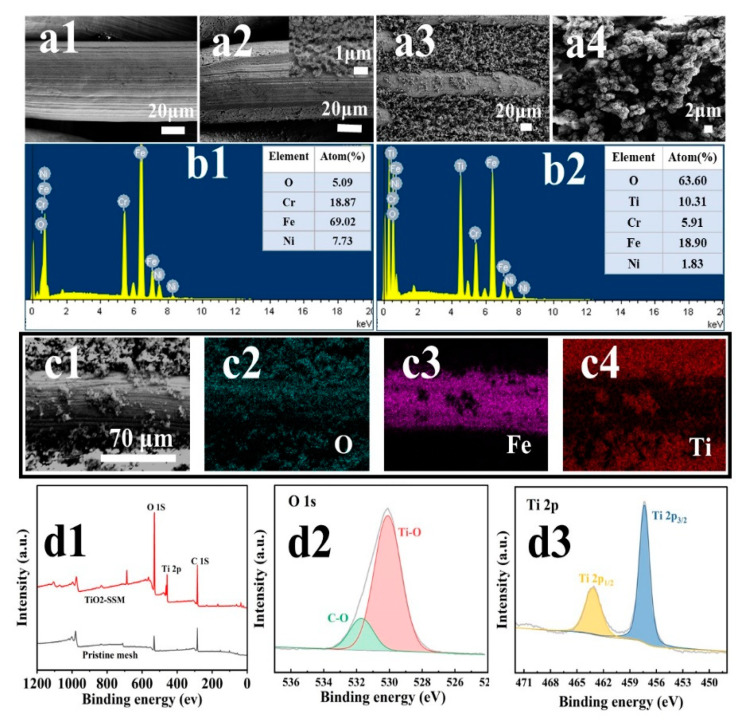
Characterization of the as-prepared SSW by SEM, EDS and XPS. SEM images (**a1**–**a4**) of TiO_2_ nanocluster-based mesh at different magnifications. (**a1**) Pristine SSM, (**a2**) etched SSM, (**a3**) TiO_2_/SSM, (**a4**) TiO_2_ clusters at high magnification. EDS patterns of pristine SSM (**b1**), EDS patterns of TiO_2_-coaded SSM (**b2**). Element (O, Fe and Ni) mapping spectra of TiO_2_-loaded SSM (**c1**–**c4**). XPS spectra of pristine SSM and TiO_2_-coaded SSM (**d1**–**d3**): (**d1**) full spectrum; (**d2**) Ti 2p peak fitting results; (**d3**) O 1s peak fitting results.

The SEM of the pristine SSM is shown in Figure 1(a1), being smooth and unattached. After being corroded by hydrofluoric acid, it became rough and porous Figure 1(a2), and the TiO_2_-coated SSM fabricated via the solvothermal reaction was observed by means of SEM Figure 1(a3), and the TiO_2_ growing on the SSM substrate fully filled the mesh pores and covered the entire surface. It was clearly seen that TiO_2_ nanoclusters with a diameter of about 2 μm had formed at a high magnification Figure 1(a4). This resultant micro–nano composite structure has a direct impact on both the surface wettability and separation ability for the as-prepared SSWs. Figure 1(b1,b2) show the EDS plots of the pristine SSM and the TiO_2_/SSM. Compared with the pristine SSM (Figure 1(b1)), the elemental contents of Fe, Cr and Ni (Figure 1(b2)) on the surface of the TiO_2_/SSM were significantly lower and the O content was evidently higher, while the appearance of the element Ti (10%) indicated the generation of TiO_2_ on the surface of the SSM. XPS was performed on the pristine SSM and TiO_2_-coated SSM, and the elemental composition and atomic valence state of the metal oxides loaded on the surface of the SSM could be reflected in the XPS spectra (Figure 1(d1–d3)). Compared with the pristine SSM, the TiO_2_-loaded SSM has more intensive peaks of elements O, Ti and C, as seen in Figure 1(d1). It can be seen that the binding energy 458.02 eV is the characteristic peak of Ti 2P, while the binding energies 284.81 eV and 530.08 eV belong to C 1s and O 1s, respectively. Valence analysis shows that the O 1s is divided into two peaks Figure 1(d2) at 529.8 eV and 532.9 eV corresponding to the C-O and Ti-O bonds, respectively. This indirectly proves that the surface of the SSM is almost covered by TiO_2_. Moreover, the two peaks of Ti 2p3/2 and Ti 2p1/2 (Figure 1(d3)) are located at 457.5 eV and 463.3 eV, indicating that at this time, Ti ions are in the 4-valent state. We carefully scraped the coating on the as-prepared SSW surface and collected the powders for the characterization by FTIR. The resultant FTIR spectra demonstrated the successful attachment of TiO_2_ onto the SSW surface (see Figure S1). The obtained results are consistent with the previous literature [18,19,20].

### 3.2. Surface Wettability of the SSM before and after Modification

**Figure 2:**
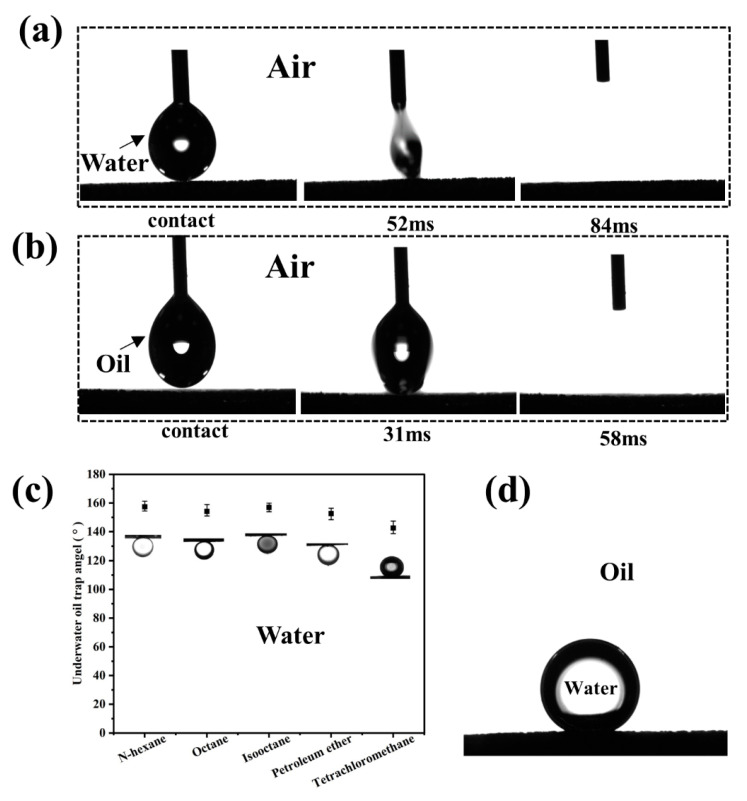
Surface wettability of the TiO_2_-coaded SSM: (**a**) instantaneous dynamics of a water droplet in air coming into contact with the mesh surface; (**b**) instantaneous dynamics of an oil droplet in air coming into contact with the mesh surface; (**c**) underwater OCA of hexane, n-octane, isooctane, petroleum ether, and carbon tetrachloride; (**d**) WCA under oil (n-hexane).

The obtained mesh film is superhydrophilic and superoleophilic in air, and both water and oil droplets can spread instantly on its surface. As can be seen in Figure 2a, the water droplet can completely spread out within 84 ms after coming into contact with the surface of the mesh film. Likewise, oil droplets can spread completely within 58 ms after coming into contact with the surface of the mesh film, as shown in Figure 2b. In the water environment, the modified SSM exhibits superoleophobicity, and the OCA is higher than 150° for hexane, n-octane, isooctane, petroleum ether, and carbon tetrachloride (Figure 2c), and the WCA in the oil environment can be as high as 158° (Figure 2d).

### 3.3. Separation of Immiscible Mixtures of Light Oil–Water and Heavy Oil–Water

**Figure 3:**
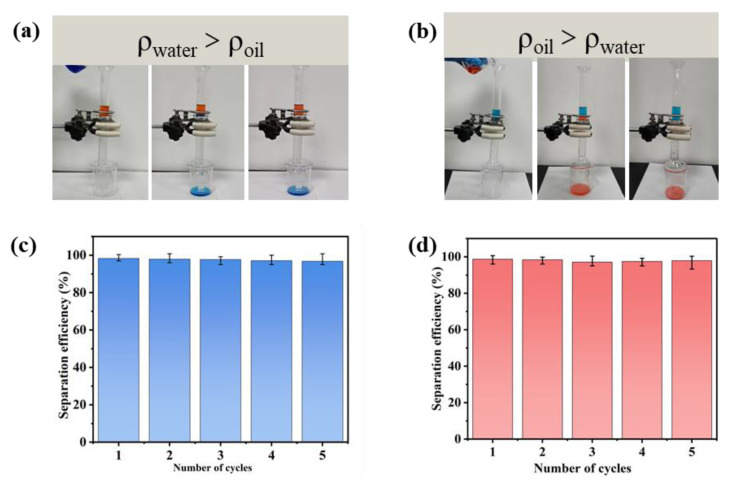
(**a**) Process of separation of light oil, (**b**) process of separation of heavy oil, (**c**) separation efficiency of light oil/water mixture for 5 cycles, (**d**) separation efficiency of heavy oil/water mixture for 5 cycles.

The prepared TiO_2_-coaded SSM can be used for the separation of light oil/water and heavy oil/water mixtures conveniently because of its specific wettability with underwater oleophobicity and underoil hydrophobicity at the same time. For the mixture separation of oil/water and emulsion, the separation device was assembled by fixing the as-prepared SSM between two 16 mm diameter glass tubes with clamps, and then fixing the device vertically on an iron stand with clamps (Figure 3a,b). The above-prepared mixtures were poured through the upper glass tube opening, and a beaker was placed below to collect the runoff filtrate.

Here, n-hexane and carbon tetrachloride were used to represent light oil and heavy oil as the oil phase (dyed red with Sudan III) and deionized water was used as the water phase (dyed blue with methylene blue), and the oil–water mixture was prepared by mixing according to V_oil_/V_water_ = 1/1. When separating n-hexane/water in Figure 3a, the mesh was wetted with water in advance (ρ_water_ > ρ_oil_), and n-hexane with the density lower than water could not pass through the mesh, but the blue-dyed water was quickly transferred to the beaker below (see Video S1). When separating the heavy oil (ρ_water_ < ρ_oil_), as shown in Figure 3b, the mesh film was wetted with carbon tetrachloride in advance, and the water could not pass through the mesh film, but the red-dyed heavy oil of CCl4 penetrated through the SSW to the beaker below quickly (see Video S2). After the separation was completed, the mesh was washed with a small amount of deionized water and ethanol consequentially, dried at room temperature, and then the separation process for light oil/water and heavy oil/water was carried out, respectively. The separation efficiency was maintained above 98% for both “oil-blocking” and “water-blocking” type membranes. Also, the separation efficiency for both membranes did not significantly decrease after five cycles, as shown in Figure 3c,d, which demonstrated the excellent recyclability of the SSMs.

The SSWs were fabricated with hierarchical micro/nano-scale architectures by means of a simple one-pot solvothermal method. Due to the specific surface wettability of superoleophobicity under water and superhydrophobicity under oil, the resultant SSW can separate both types of light and heavy oil/water effectively and efficiently, driven solely by gravity.

### 3.4. Emulsion Separation of Oil-in-Water and Water-in-Oil

**Figure 4:**
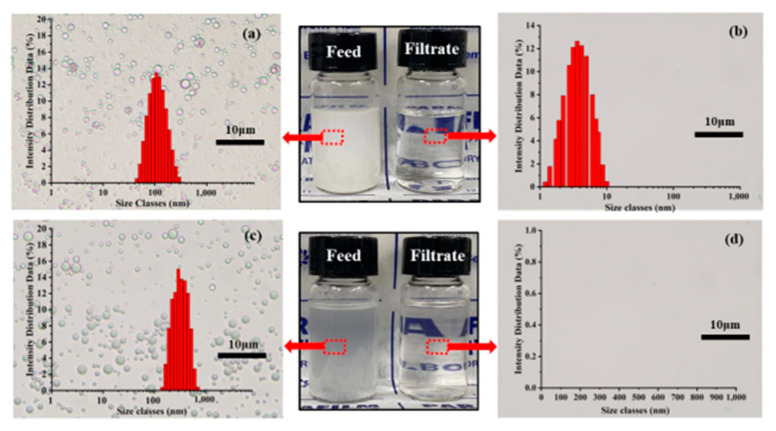
Optical photos of emulsion feed and filtrate, droplet size distribution, and color comparison of appearance. (**a**) water/isooctane (w/o) emulsion before separation, (**b**) water/isooctane (w/o) emulsion after separation; (**c**) isooctane/water (o/w) emulsion before separation, (**d**) isooctane/water (o/w) emulsion after separation.

The as-prepared SSM is also desirable for the separation of both water-in-oil emulsions (a) and oil-in-water emulsions (c) in Figure 4**.** The liquid appeared milky white before emulsion separation, with water or oil droplets ranging in size from dozens to hundreds of nanometers (Figure 4). Compared to the original milky feeds before filtration, the collected filtrates were transparent and colorless after filtration, as clearly recorded using an optical microscope (see Videos S3 and S4). For the water-in-oil emulsion, the water droplet size decreased from hundreds of nanometers to 10 nm (Figure 4a,b). Also, for the oil-in-water emulsions, the oil droplet size was several hundred nanometers before filtration (Figure 4c), but almost no oil droplets were found in the separated liquids (Figure 4d).

Compared to other membranes that have been reported with relatively few separation targets, we chose four typical separation targets, all of which were well separated under simple gravity and had similar separation performance (see Table 1).

## 4. Conclusions

In this study, the excellent superwetting materials were fabricated using one-pot solvothermal synthesis and applied for versatile separation of immiscible and emulsified oil–water mixtures. Satisfactory separation results were obtained for the selected typical targets including immiscible light oil–water mixtures, heavy oil–water mixtures, oil-in-water emulsions and water-in-oil emulsions under solely gravity. The as-prepared superamphiphilic SSMs exhibited excellent separation efficiency greater than 98% for oil/water separation and 99.0% for emulsion separation, respectively. Compared with the previous membranes, it provided a facile and simple separation strategy for complex oil/water mixtures, which was beneficial for versatile application in water treatment.

## Figures and Tables

**Table 1:** The comparison of different membrane materials for the emulsion separation.

Materials	Method	Wettability	Separation Target	Driving Force	Separation Efficiency	Ref.
TiO_2_@Cu mesh	Solvothermal method	Superamphiphilic	Water-in-oil Oil-in-water	Gravity	99.97%	[21]
SiO_2_/PDMS composite	Immersing	Superhydrophobic	Water-in-oil	Gravity	>99.4%	[22]
Cellulose sponge	Dissolution and regeneration method	Superoleophobicity under water	Oil-in-water	Gravity	>99.2%	[23]
C@SiO_2_@SSM	Spraying	Superhydrophobic	Oil/water separation	Gravity	>99.0%	[24]
Copper mesh	In situ growth of MOF	Underwater superoleophobicity and underoil superhydrophobicity	Oil/water separation	Gravity	>97%	[25]
HDMS@SiO_2_@SSM	Spraying	Asymmetric wettability	Water-in-oil Oil-in-water	Gravity 4 Kpa	>98.6% >97.5%	[26]
TiO_2_@SSM	Solvothermal method	Superamphiphilic	Oil/water separation Water-in-oil Oil-in-water	Gravity Gravity Gravity	>98% >99.0% >99.0%	This work

## Data Availability

The data are available from the corresponding author.

## Supplementary Materials

The following supporting information can be downloaded at: https://www.mdpi.com/article/10.3390/membranes13100808/s1, Figure S1: FTIR spectra of TiO_2_ NPs scraped from the SSM surface; Video S1: The as-prepared SSM membrane was used to separate immiscible n-hexane/water; Video S2: The as-prepared SSM membrane was used to separate immiscible n-water/CCl_4_; Video S3: The as-prepared SSM membrane was used to separate o/w emulsion; Video S4: The as-prepared SSM membrane was used to separate w/o emulsion.
