# Supplementary material for: Superwetting Stainless Steel Mesh Used for Both Immiscible Oil/Water and Surfactant-Stabilized Emulsion Separation
Source: Membranes (Basel). 2023 Sep 24;13(10):808. doi: 10.3390/membranes13100808 (PMC10608510; doi:10.3390/membranes13100808)
Supplement: Supplementary file 1 [file membranes-13-00808-s001.zip › membranes-2571694-supplementary.pdf]

membranes

MDPI

Supporting file:

Superwetting Stainless Steel Mesh Used for Both Immiscible Oil/water and Surfactant-stabilized Emulsion Separation

Yu-Ping Zhang [1,2]*, Ya-Ning Wang [2], Li Wan [1], Xin-Xin Chen [3] and Chang-Hua Zhao [2]

[1]College of Chemistry and Materials Engineering, Hunan University of Arts and Science, Changde 415000, China
[2]College of Chemistry, Zhengzhou University, Zhengzhou 450001, China
[3]College of Chemistry and Chemical Engineering, Henan Institute of Science and Technology, Xinxiang 453003, China
* Correspondence: beijing2008zyp@163.com

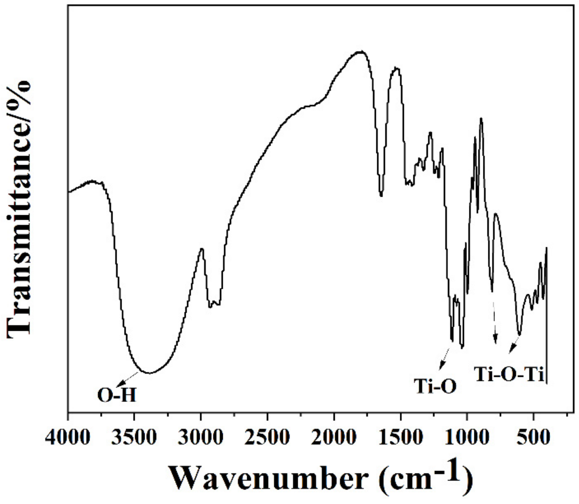

Figure S1. FTIR spectra of TiO_2 NPs scraped from the SSM surface.

Fig. S1 shows the FTIR spectra of TiO2 NPs to be in the range of 4000–400 cm^{-1}. The spectrum with a broad band at 3400 cm^{-1} is attributed to the surface hydroxyl group. The peaks around 1200 cm^{-1} might be attributed to the presence of stretching vibrations of Ti-O bonds. The band at 500–700 cm^{-1} is attributed to the Ti-O-Ti stretching vibrations, which demonstrated the successful attachment of TiO_2 on the SSW surface.

When separating n-hexane/water, the mesh was wetted with water in advance ($\varrho_{water} > \varrho_{oil}$), and n-hexane with the density lower than water could not pass through the mesh, but the blue-dyed water was quickly transfer to the beaker below (see **Video.S1**).

When separating the heavy oil ($\varrho_{water} < \varrho_{oil}$), the mesh film was wetted with carbon tetrachloride in advance, and the water could not pass through the mesh film, but the red-dyed heavy oil of CCl_4 penetrated through the SSW to the beaker below quickly (see **Video.S2**).

Viedo.S1 The as-prepared SSM membrane was used to separate immiscible n-hexane/water.

Viedo.S2 The as-prepared SSM membrane was used to separate immiscible n-water/CCl_4.

Viedo.S3 The as-prepared SSM membrane was used to separate O/W emulsion.

Viedo.S4 The as-prepared SSM membrane was used to separate W/O emulsion.
