# Supplementary figures and images for: Superwetting Stainless Steel Mesh Used for Both Immiscible Oil/Water and Surfactant-Stabilized Emulsion Separation
Source: Membranes (Basel). 2023 Sep 24;13(10):808. doi: 10.3390/membranes13100808 (PMC10608510; doi:10.3390/membranes13100808)

## Slide 1
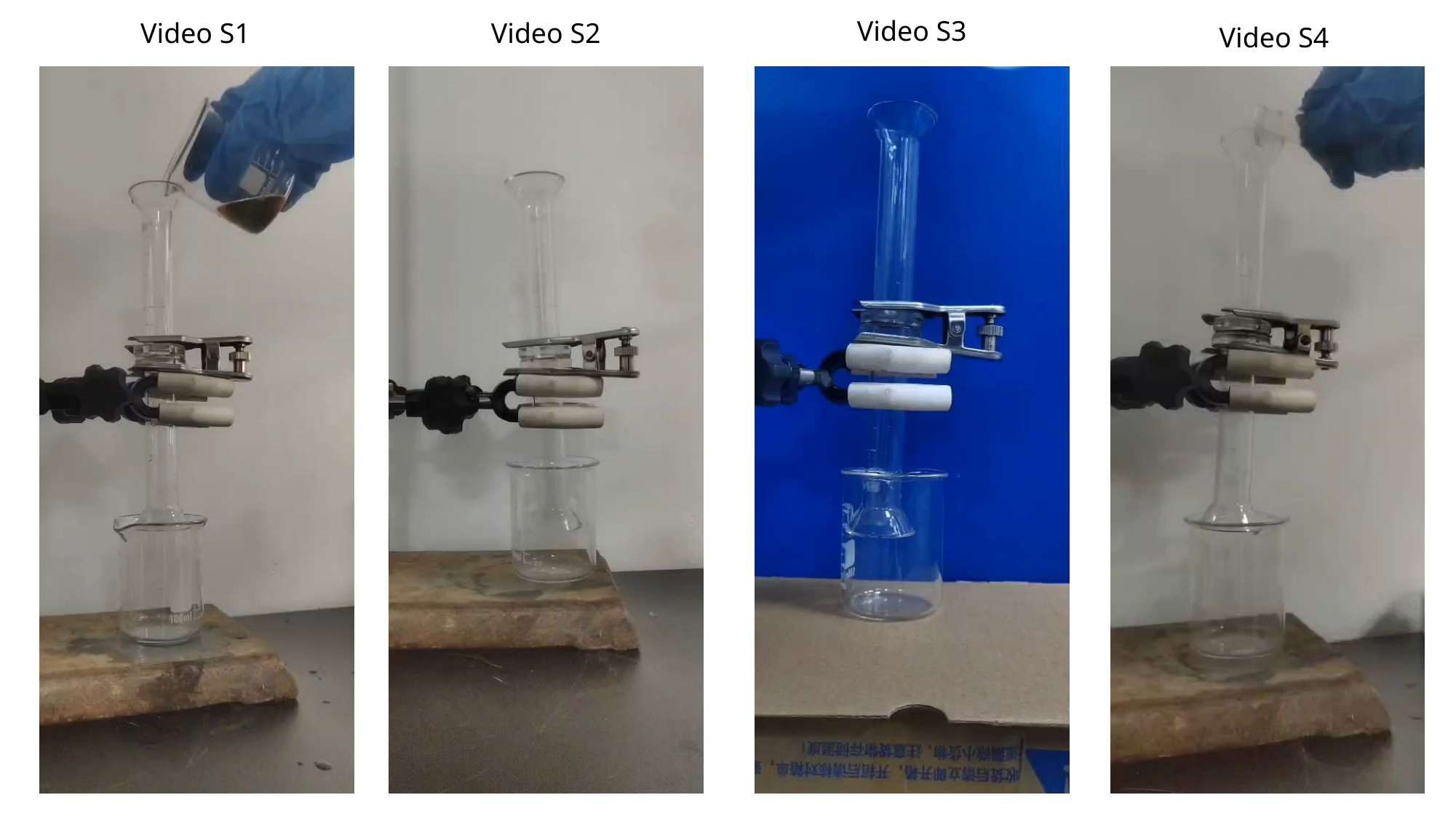

Video S3
Video S1
Video S2
Video S4

Supplement: Supplementary file 1 [file membranes-13-00808-s001.zip › Videos.pptx]
